# Metformin-Induced Lactic Acidosis: A Case Study

**DOI:** 10.7759/cureus.2152

**Published:** 2018-02-04

**Authors:** Waliul Chowdhury, Muhammad Uzair Lodhi, Intekhab Askari Syed, Umer Ahmed, Maxwell Miller, Mustafa Rahim

**Affiliations:** 1 Medical Student, Department of Medicine, Raleigh General Hospital, Beckley, Wv; 2 Research Associate, Broad Institute of Mit and Harvard; 3 Medical Student, Department of Medicine, Lincoln Memorial University-Debusk College of Osteopathic Medicine; 4 Assistant Clinical Professor of Internal Medicine, West Virginia University School of Medicine

**Keywords:** metformin, lactic acidosis, metformin overdose

## Abstract

Metformin is the first line management for patients with Type 2 diabetes mellitus. Metformin-induced lactic acidosis (MALA) is a severe side effect of metformin in high doses. However, there have not been many reported cases of MALA. The threshold metformin concentration needed to induce lactic acidosis is still not fully understood. It is important for physicians to measure metformin levels upon admission in Type 2 diabetes patients who take metformin and present with suspected lactic acidosis. We present a case of a 40-year-old Caucasian male who presented with severe lactic acidosis shortly after overdosing on metformin.

## Introduction

Metformin works as an antihyperglycemic agent and promotes euglycemia. However, metformin can exacerbate hypoglycemia in patients who take other glucose-lowering drugs, such as sulfonylureas [1-2]. The major side effect of metformin is lactic acidosis [1-5]. There are few cases reported on Type 2 diabetes patients presenting with lactic acidosis where metformin was the major inducing factor. We will present a 40-year-old male patient who overdosed on metformin and developed lactic acidosis the following day. 

## Case presentation

A 40-year-old Caucasian male smoker presented with malaise. He mentioned feeling like this for the past three months but not as severe as when he was admitted. He did not mention any chest pain, heaviness, or pressure. He denied fever, chills, diarrhea, constipation, abdominal pain, nausea, or vomiting. He also denied odynophagia, dysphagia, hematemesis, hematochezia, hematuria, or dysuria. He did not mention having any rashes, discoloration, or depigmentation of the skin. He denied any alcohol or substance abuse. His past medical history included: Type 2 diabetes mellitus, bipolar disorder, chronic obstructive pulmonary disease, gastroesophageal reflux disease, and arthritis. His past surgeries included a cholecystectomy and a right-sided ankle fusion. His home medications included metformin (1,000 mg twice a day), acetaminophen (as needed), amlodipine (5 mg once daily), famotidine (20 mg twice a day), gabapentin (100 mg three times a day), glipizide (5 mg once a day), lurasidone (80 mg once a day), and mirtazapine (15 mg once a day).

Upon physical examination, he was not in acute distress. His vitals were as follows: afebrile, blood pressure of 178/107, heart rate of 115, respiratory rate of 20 breaths per minute, and an oxygen saturation of 96%. His pupils were equal, round, and reactive to light and accommodation. There was no pallor or icterus. Extraocular movements were normal. There was no nystagmus or strabismus. The neck was supple with no thyromegaly, lymphadenopathy, or carotid bruits. Cardiac auscultation revealed a regular rhythm without murmurs or gallops and audible S1 and S2. Respiratory sounds were clear to auscultation with no crackles, wheezes, or bronchial breath sounds. The abdomen was soft and non-tender with no visceromegaly or abnormal masses. His bowel sounds were clearly audible. His extremities showed no peripheral edema, clubbing, or cyanosis. His peripheral pulses were symmetrical and normal. 

His cardiac markers were all negative. On admission, his lactate level was 7.1 (Table 1). His metformin level was 14 mcg/mL on Day 2 of admission. The remainder of his labs, including a complete metabolic panel (Table 2), complete blood count (Table 3), and toxicology report (Table 4), is shown below.

**Table 1:** Blood Lactate Concentration

Plasma level (mmol/L)	Day of admission	Time	Reference Range (mmol/L)
7.1	Day 2	06:50	0.4 - 2.0
4.5	Day 2	13:12	0.4 - 2.0
0.4	Day 4	04:30	0.4 - 2.0

**Table 2:** Metabolic Panel

	Time	Value	Units	Reference Range
Glucose (Glu)	Day 1	188	mg/dL	70 - 99
Ammonia	Day 1	60	Umol/L	11 - 32
Chloride (Cl)	Day 1	111	mEq/L	98 - 107
Calcium (Ca)	Day 1	8.3	mg/dL	8.5 - 10.2
Albumin	Day 1	3.3	g/dL	3.5 - 5.5
Aspartate aminotransferase serum glutamic oxaloacetic transaminase (AST SGOT)	Day 1	54	U/L	10 - 40
Alanine aminotransferase serum glutamic oxaloacetic transaminase (ALT SGOT)	Day 1	37	U/L	7 - 56
Total Bilirubin	Day 1, Day 3	1.6, 1.0	mg/dL	0.2 - 1.0
Magnesium (Mg)	Day 3	2.2	mg/dL	1.6 - 2.6
Phosphate (Phos)	Day 3	4.6	mg/dL	2.5 - 4.7
Glomerular filtration rate (GFR)	Day1, Day 3	> 60, 26	mL/min/1.73 m^2^	90 - 120
Blood urea nitrogen/creatinine ratio (BUN/Cr)	Day 1, Day 3	13, 9	mmol/L	2.5 - 7.1

**Table 3:** Complete Blood Count

	Time	Value	Units	Reference Range
White blood cells (WBC)	Day 1	3.7 x 10^3^	µL	4.8 - 10.8 x 10^3^
Red blood cells (RBC)	Day 1	4.11 x 10^6^	cells/mm^3^	4.70 - 6.10 x 10^6^
Hemoglobin (HgB)	Day 1	12.5	g/dL	14.0 - 18.0
Hematocrit (Hct)	Day 1	36.9	%	42.0 - 52.0
Mean corpuscular volume (MCV)	Day 1	89.9	fl	80.0 - 94.0
Mean corpuscular hemoglobin (MCH)	Day 1	30.5	pg	27.0 - 31.0
Mean corpuscular hemoglobin concentration (MCHC)	Day 1	33.9	g/dL	33.0 - 37.0
Red cell distribution width (RDW)	Day 1	16.2	%	11.5 - 14.5
Mean platelet volume (MPV)	Day 1	9.8	fl	6.2 - 10.6
Platelet count	Day 1	68 x 10^3^	µL	130 - 400 x 10^3^
Neutrophil	Day 1	63	%	42.0 - 75.0
Lymphocyte	Day 1	21.8	%	20.0 - 40.0
Monocyte	Day 1	9.8	%	2.0 - 7.0
Basophil	Day 1	0.6	%	0.0 - 1.0
Eosinophil	Day 1	4.8	%	0.0 - 3.0
Neutrophil count	Day 1	2.4 x 10^3^	µL	1.5 - 7.1 x 10^3^

**Table 4:** Toxicology Report

Drug	Result	Reference Range
Salicylates	< 1.7 mg/dL	15 - 30 mg/dL
Acetaminophen	3 mg/mL	10 - 30 mg/mL
Alcohol	< 11 mg/dL	0 - 10 mg/dL

## Discussion

Unfortunately, his metformin levels were not measured on the first day of admission. They were measured on Day 2. Measuring his metformin level immediately when his lactic acid level was 7.1 upon admission would have given us an idea what level of metformin was associated with that level of lactic acidosis. All physicians should measure metformin levels immediately in Type 2 diabetes patients who present with signs of lactic acidosis upon admission. This would provide enough data to understand the likely threshold level of metformin required to induce lactic acidosis in patients. 

It is still being investigated whether measuring metformin and lactate levels have an effect on mortality. For example, Vecchio et al. retrospectively observed 60 patients with cases of lactic acidosis (pH < 7.35, arterial lactate > 5 mmol) associated with metformin toxicity (plasma level > 4 mcg/mL) from 2007 to 2011. All patients had an acute renal failure in addition to lactic acidosis. They showed metformin concentrations to have a significant correlation with plasma lactate levels (p = 0.001, R = 0.41). However, the mean concentration of metformin and lactate levels were not statistically significant between surviving and deceased patients [4].

Another study by Lalau et al. collected data from 49 metformin-treated patients with lactic acidosis (arterial lactate > 5 mmol/L, blood pH < 7.35) and analyzed their metformin concentrations. They also showed no significant difference in lactate levels between surviving and deceased patients. However, the mean metformin concentrations were three times higher in the patients who survived (20.6 mg/L) compared to those who died (6.3 mg/L) [5]. Both studies concluded that neither arterial lactate levels nor plasma metformin concentrations can determine the likelihood of mortality [4-5]. Instead, the extent of underlying comorbid conditions had more of a significant prognostic value [4-5].

The patient also admitted to taking excess amounts of acetaminophen. This contradicts the initial lab analysis, which showed his acetaminophen levels at 3 mg/mL. Subsequent analysis showed his serum acetaminophen to be 54 mg/mL (reference range = 10 - 30 mg/mL). The initial discrepancy was most likely a lab error.

Acetaminophen is well known for causing liver injury when taken in toxic amounts [6]. Acetaminophen-induced hepatotoxicity occurs through the formation of the metabolite N-acetyl-para-benzo-quinone imine (NAPQI), which depletes glutathione stores. This causes oxidative stress and damage to the liver's mitochondria, which inhibits the body's ability to produce adenosine triphosphate (ATP). NAPQI also binds to cellular proteins, such as mitochondrial proteins. This decreases important antioxidant functions and disrupts the mitochondrial ATP synthase alpha subunit, which all deplete ATP formation. The end result is the formation of centrilobular necrosis of the liver [6].

Acetaminophen-induced liver damage could play a role in exacerbating the effects of MALA. This is because of lactate produced by muscle cells via anaerobic glycolysis shifts to the liver. The liver acts by converting the lactic acid into glucose via gluconeogenesis [7]. If there is liver damage, gluconeogenesis could be affected causing a build-up of a lactic acid substrate in the body.

Intravenous (IV) hydration was successfully used to control the lactic acidosis. However, other treatments are available. For example, a recent study by Oyaizu-Toramaru et al. investigated the use of targeting oxygen-sensing prolyl hydroxylase (PHD) in patients with metformin-induced lactic acidosis. They found that inhibiting PHD activates a transcription factor, known as hypoxia-inducible factor (HIF), which promotes lactate efflux and gluconeogenesis by using lactate as the substrate; these all lower circulating lactate levels [8]. Even though there are many more treatment options for lactic acidosis, IV hydration therapy was effective in this case.  

## Conclusions

Lactic acidosis is a well-known side effect of metformin. However, monitoring lactate and metformin levels have not yet been proven to improve the patient’s prognosis. Physicians should make it part of their protocol to measure metformin levels in Type 2 diabetics who present with signs and symptoms of possible lactic acidosis. When presented with a case of MALA, IV hydration therapy should be sufficient therapy if diagnosed promptly. Although MALA is rare, it is still possible, as seen in this patient's case, and physicians should be prepared for prompt diagnosis and treatment.
